# Renal AA amyloidosis leading to early diagnosis and treatment of takayasu arteritis: a case report and review of the literature

**DOI:** 10.1007/s00392-020-01655-4

**Published:** 2020-04-28

**Authors:** Igor Kos, Stephan Stilgenbauer, Moritz Bewarder

**Affiliations:** Internal Medicine I, Saarland University Medical Center, Homburg, Germany

**Keywords:** AA amyloidosis, Takayasu arteritis, Proteinuria, Tocilizumab

Sirs:

A 31-year-old female patient from India presented to our institution with proteinuria. The initial screening by dipstick urinalysis showed a proteinuria level of 1000 mg/dL, while the 24-h urine collection registered 4.01 g of protein and 602 mg of creatinine. These abnormalities were diagnosed during her second pregnancy and persisted for 5 months after delivery. Retrospectively, proteinuria was already present during her first pregnancy 2 years before but was not followed up thereafter. Apart from occasional mild shoulder pain, the patient reported no complaints, in particular, no B-symptoms and Raynaud’s phenomenon was absent. The physical examination was unremarkable, there was no edema or dermatological alterations, and all peripheral pulses were palpable.

Due to the unclear origin of the nephrotic syndrome, a biopsy of the kidney was performed. The histological results showed vascular and glomerular AA amyloidosis, which was also verified by high levels of serum amyloid A (48 mg/dl—reference value < 10 mg/dl). An echocardiogram and abdominal ultrasound were performed to exclude further organ involvement but were unremarkable.

To investigate the underlying causes of AA amyloidosis, we performed an extensive microbiological panel, which showed negative results for viruses [hepatitis B/C, human immunodeficiency virus (HIV), Epstein-Barr virus (EBV), Cytomegalovirus (CMV)], bacteria (specific tests for *Treponema pallidum*, *Yersinia* and mycobacteria, negative blood cultures) and parasites (*Leishmania*, *Schistosoma*, *Cryptosporidium*, *Giardia*, *Amoeba*). The diagnostic workup for rheumatological diseases did not reveal any anomalies, with normal titers of rheumatoid factor, anti-cyclic citrullinated peptide antibodies, anti-ds-DNA antibodies, anti-nuclear antibodies and anti-neutrophil cytoplasmic antibodies. Due to an erythrocyte sedimentation rate (ESR) of 80 mm/h and a C-reactive protein (CRP) of 30 mg/l, the search for an inflammatory trigger was continued with a colonoscopy, which was also unremarkable. The bone marrow biopsy showed an infiltration of 10–15% polyclonal plasma cells compatible with chronic inflammation but with no signs of a specific diagnosis such as hemophagocytosis, lymphoma or leukemia.

Due to conspicuous hypochromic and microcytic anemia, we performed hemoglobin electrophoresis, which confirmed the diagnosis of beta-thalassemia minor. However, this condition is not commonly associated with AA amyloidosis and was unlikely to be the underlying cause but rather an incidental finding.

With no specific results thus far, we performed an FDG-positron emission tomography (PET) scan, which showed mild inflammation of the aorta and carotid arteries (Fig. 1a, b). These results were indicative of large-vessel vasculitis. Considering the patient’s characteristics and clinical presentation, the diagnosis of Takayasu arteritis (TA) was made.

**Fig. 1:**
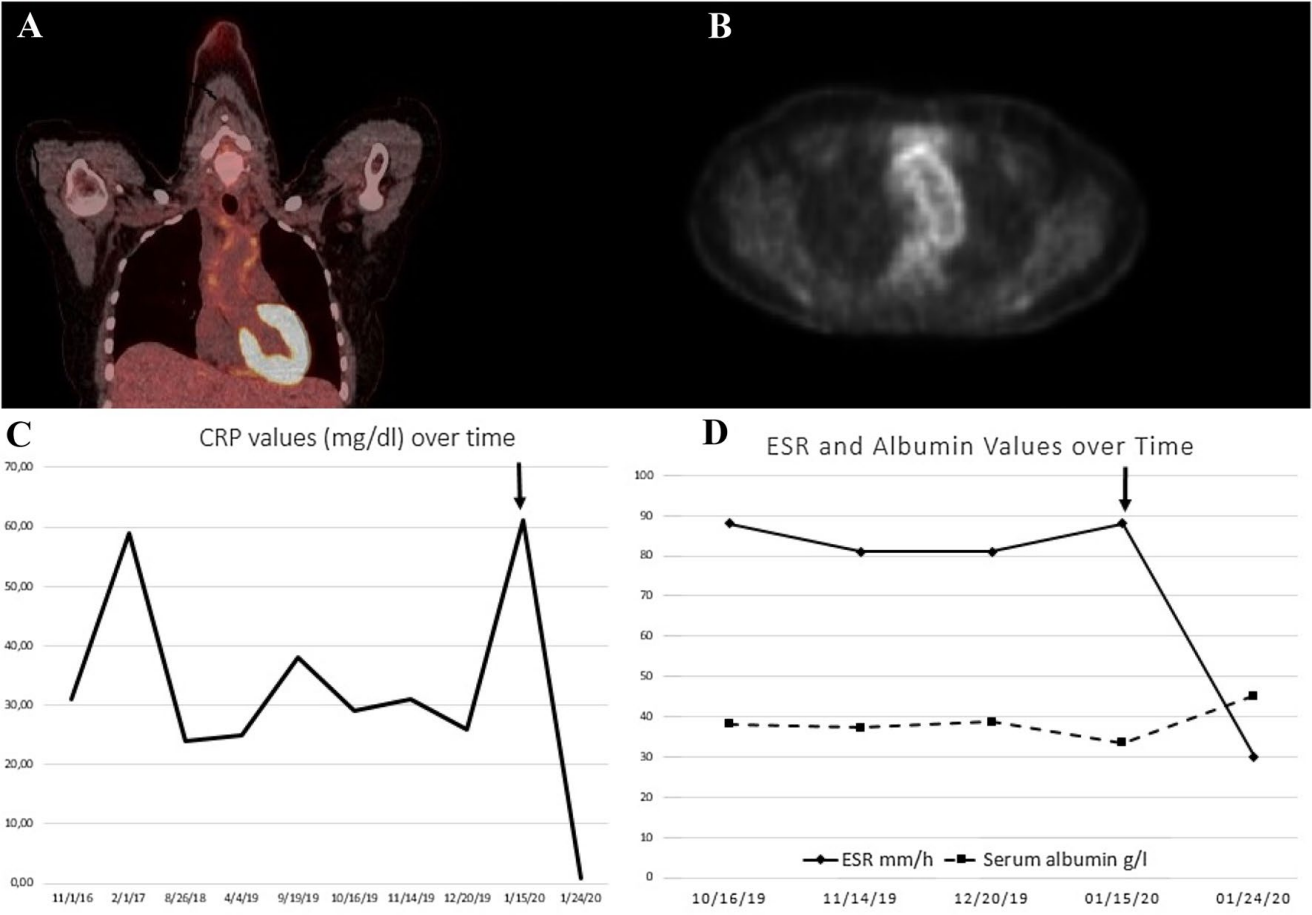
**a** FDG-PET CT scan showing increased metabolic activity in the aortic arch. **b** FDG-PET CT scan showing increased metabolic activity of the aortic wall. **c** Shows the values of CRP over time. **d** Shows the values of serum albumin and ESR over time. The arrows represents the introduction of therapy

Treatment with subcutaneous methotrexate 15 mg/week and prednisone 30 mg/day was initiated. Subcutaneous tocilizumab (162 mg) was added to the initial therapy, allowing for steroid tapering. Two weeks after the start of therapy, we observed a quick normalization of all inflammatory parameters (CRP: 0.8 mg/dL and ESR: 30 mm/h) and proteinuria and an increase in serum albumin levels (from 33.5 to 45.1 g/L). Figure 1c, d summarizes the course of CRP, ESR and serum albumin over time. A second renal biopsy was not performed.

Amyloidoses are a group of diseases characterized by the accumulation of pathological amyloids in different tissues [1]. The most common types of amyloidoses are amyloid A amyloidosis (AA), light chain amyloidosis (AL), transthyretin amyloidosis (ATTR) and β2-microglobulin amyloidosis (Aβ2M). The symptoms are heterogeneous and can vary from carpal tunnel syndrome to chronic cardiac failure [2, 3]. The heart, kidneys, gastrointestinal tract and nervous system are frequently impaired, and without treatment, the disease can lead to a loss of function and mortality [4]. The prognosis remains poor, especially in patients with cardiac involvement [5]; however, an early diagnosis is of fundamental importance, since an appropriate therapy can, depending on the amyloidosis type, reduce or reverse its progress [6, 7].

Renal involvement is the most common presentation of AA amyloidosis and is found in approximately 90% of patients. The involvement can vary from mild proteinuria to renal failure or nephrotic syndrome. While in developing countries, the most common cause of AA amyloidosis is infections, in western countries, it is commonly associated with autoimmune diseases [8]. Rheumatoid arthritis, juvenile idiopathic arthritis and ankylosing spondylitis are the most frequent underlying diseases associated with AA amyloidosis, accounting for 23–51%, 7–48% and 0–12% of all cases, respectively [9]. The initial suspicion that the underlying cause was likely to be of infectious origin was excluded after an extensive microbiological investigation. Therefore, we initiated a search for other inflammatory diseases that lead to the diagnosis of TA. Even though only one diagnostic criterion of the American College of Rheumatology (ACR) for TA was met at the time of diagnosis (age under 40 years), the totality of evidence was still very suggestive. Other causes of aortitis, such as syphilis or other acute infections, were ruled out using immune assays and blood cultures. Furthermore, the elevated inflammatory parameters over more than 2 years were also suggestive of a chronic process and did not favor other acute infections. Although the ACR criteria are an important diagnostic tool, they include late features of the disease and thus advanced vascular involvement. Considering the early diagnosis in our patient, these changes had not yet taken place, making the diagnosis with the application of the ACR criteria impossible. However, FDG-PET/CT imaging has a strong sensitivity and specificity for the diagnosis of TA, and due to the highly suggestive findings in this case, we rely on imaging results rather than vascular symptoms for making the diagnosis of TA.

The association between TA and AA amyloidosis has been described, but due to the low incidence of both conditions, only a few cases are reported in the literature [8]. Table 1A shows the most common causes of AA amyloidosis, while Table 1B shows the prevalence of TA in different countries.

**Table 1:** **a** Prevalence and incidence of TA differ greatly between countries with the highest reported prevalence in Japan and the lowest in the USA. **b** Underlying causes for AA amyloidosis are predominantely either of infectious or autoimmune origin but can also include malignant diseases

Country	Prevalence (per million)	Incidence (per million per year)
(A) Prevalence and incidence of takayasu arteritis worldwide* [8]		
Japan	40	1–2
United Kingdom	4.7	0.8
Turkey	14.7–33	0.38–3.4
Northern Europe	6.4–22	0.4–1.5
Kuwait	7.8	2.2
USA	0.9	2.6
(B) Causes of AA amyloidosis with strong association [1, 3]		
Chronic arthritis		
Bronchiectasis		
IV-drug abuse		
Osteomyelitis		
Tuberculosis/leprosy		
Crohn disease		
Vasculitis		
Familial mediterranean fever		
Neoplasia		
Castleman disease		

*This table reflects data of several epidemiological studies with different methods and diagnostic criteria [8]

The time of the manifestation of AA amyloidosis after the diagnosis of the underlying disease can vary. Studies investigating the development of AA amyloidosis in rheumatological patients have reported a median of 17–26 years of disease before the onset of amyloidosis. It is rather unusual that AA amyloidosis manifests itself as the initial symptom of the underlying disease [10].

The presentation of Takayasu arteritis is unspecific, and approximately 10% of all cases can be asymptomatic. Clinical manifestations can vary from constitutional to dermatological symptoms; therefore, in many patients, TA is diagnosed only once vascular involvement is already advanced.

Few reports have demonstrated an association between AA amyloidosis and Takayasu arteritis [8, 11, 12], and only in a few cases was AA amyloidosis the initial manifestation of TA [11, 12]. Considering the slow progression and sometimes long periods of subclinical disease observed in TA, it is reasonable to suggest that AA amyloidosis, in this scenario, shows a different natural history when compared to other diseases, such as rheumatoid arthritis, in which the clinical manifestations and diagnosis are present for many years before the onset of AA amyloidosis.

The choice of adding tocilizumab despite pending EMA approval has a strong rationale due to the positive effects of tocilizumab on both conditions. Retrospective studies and case reports have demonstrated good results with tocilizumab in AA amyloidosis, including the suppression of SAA levels and the regression of AA protein deposits [13]. The use of IL-6 inhibition alone in TA has also been demonstrated to be effective, but the evidence is still limited [14]. Furthermore, IL-6 blockade in patients with rheumatic diseases is associated with an improvement of vascular function [15]. Considering the severe manifestations of AA amyloidosis in our patient, the first goal of therapy was the reduction of proteinuria and the prevention of further renal damage. The introduction of tocilizumab in this patient resulted in a quick reduction of all inflammatory parameters and proteinuria. Considering the very early start of therapy in the course of vasculitis, it is reasonable to expect disease control with a clinically significant reduction in progression, preventing advanced vascular involvement.

## Data Availability

All data is available and can be provided if requested.
